# Diversity of ABC transporter genes across the plant kingdom and their potential utility in biotechnology

**DOI:** 10.1186/s12896-016-0277-6

**Published:** 2016-05-31

**Authors:** Thomas S. Lane, Caroline S. Rempe, Jack Davitt, Margaret E. Staton, Yanhui Peng, Douglas Edward Soltis, Michael Melkonian, Michael Deyholos, James H. Leebens-Mack, Mark Chase, Carl J. Rothfels, Dennis Stevenson, Sean W. Graham, Jun Yu, Tao Liu, J. Chris Pires, Patrick P. Edger, Yong Zhang, Yinlong Xie, Ying Zhu, Eric Carpenter, Gane Ka-Shu Wong, C. Neal Stewart

**Affiliations:** The Graduate School of Genome Science and Technology, University of Tennessee, Knoxville, TN 37996 USA; Department of Entomology and Plant Pathology, University of Tennessee, Knoxville, TN 37996 USA; Department of Plant Sciences, University of Tennessee, Knoxville, TN 37996 USA; Department of Biology, University of Florida, Gainesville, FL 32611 USA; Florida Museum of Natural History, Gainesville, FL 32611 USA; Genetics Institute, University of Florida, Gainesville, FL 32611 USA; Department of Biology, Cologne Biocenter, University of Cologne, Cologne, 50674 Germany; Department of Biology, University of British Columbia, Kelowna, BC V1V 1V7 Canada; Department of Biological Sciences, University of Georgia, Athens, GA 30602 USA; Jodrell Laboratory, Royal Botanic Gardens Kew, Richmond, Surrey, TW9 3AE UK; Department of Biology, Duke University, Durham, NC 27708 USA; Department of Integrative Biology, University of California, University Herbarium, Berkeley, CA 94720-2465 USA; New York Botanical Garden, Bronx, New York, NY 10458 USA; Department of Botany, University of British Columbia, Vancouver, BC Canada; CAS Key Laboratory of Genome Sciences and Information, Beijing Key Laboratory of Genome and Precision Medicine Technologies, Beijing Institute of Genomics, Chinese Academy of Sciences, Beijing, 100101 People’s Republic of China; College of Marine Life Sciences, Ocean University of China, Qingdao, 266003 People’s Republic of China; Bond Life Sciences Center, Division of Biological Sciences, University of Missouri, Columbia, MO USA; BGI-Shenzhen, Beishan Industrial Zone, Yantian District, Shenzhen, 518083 China; Department of Computer Science, HKU-BGI Bioinformatics Algorithms and Core Technology Research Laboratory, University of Hong Kong, Pokfulam, Hong Kong; School of Bioscience and Bioengineering, South China University of Technology, Guangzhou, China; Department of Biological Sciences, University of Alberta, Edmonton, AB T6G 2E9 Canada; Department of Medicine, University of Alberta, Edmonton, AB T6G 2E1 Canada

**Keywords:** ABC transporter, Transcriptomics, Computational biology, Taxonomic diversity

## Abstract

**Background:**

The ATP-binding cassette (ABC) transporter gene superfamily is ubiquitous among extant organisms and prominently represented in plants. ABC transporters act to transport compounds across cellular membranes and are involved in a diverse range of biological processes. Thus, the applicability to biotechnology is vast, including cancer resistance in humans, drug resistance among vertebrates, and herbicide and other xenobiotic resistance in plants. In addition, plants appear to harbor the highest diversity of ABC transporter genes compared with any other group of organisms. This study applied transcriptome analysis to survey the kingdom-wide ABC transporter diversity in plants and suggest biotechnology applications of this diversity.

**Results:**

We utilized sequence similarity-based informatics techniques to infer the identity of ABC transporter gene candidates from 1295 phylogenetically-diverse plant transcriptomes. A total of 97,149 putative (approximately 25 % were full-length) ABC transporter gene members were identified; each RNA-Seq library (plant sample) had 88 ± 30 gene members. As expected, simpler organisms, such as algae, had fewer unique members than vascular land plants. Differences were also noted in the richness of certain ABC transporter subfamilies. Land plants had more unique ABCB, ABCC, and ABCG transporter gene members on average (*p* < 0.005), and green algae, red algae, and bryophytes had significantly more ABCF transporter gene members (*p* < 0.005). Ferns had significantly fewer ABCA transporter gene members than all other plant groups (*p* < 0.005).

**Conclusions:**

We present a transcriptomic overview of ABC transporter gene members across all major plant groups. An increase in the number of gene family members present in the ABCB, ABCC, and ABCD transporter subfamilies may indicate an expansion of the ABC transporter superfamily among green land plants, which include all crop species. The striking difference between the number of ABCA subfamily transporter gene members between ferns and other plant taxa is surprising and merits further investigation. Discussed is the potential exploitation of ABC transporters in plant biotechnology, with an emphasis on crops.

**Electronic supplementary material:**

The online version of this article (doi:10.1186/s12896-016-0277-6) contains supplementary material, which is available to authorized users.

## Background

The ATP-binding cassette (ABC) transporter family is one of the largest known protein superfamilies in biology, which is represented in all living organisms [1–7]. Shared among members within the ABC transporter protein superfamily is the ability to hydrolyze adenosine triphosphate (ATP), which is used in a wide array of functions, including DNA repair, RNA translocation, and most commonly, active transport of a wide variety of substrates across various types of membranes in cells [3].

ABC transporters have three structural types. Full transporters are composed of two transmembrane domains (TMD) and two nucleotide-binding domains (NBD). Half transporters are composed of one TMD and one NBD, which dimerize in pairs to create virtual full transporters as homodimers or heterodimers. A third type of transporter has no TMDs but two NBDs [5]. The NBD is present in all three structural types and contains many key conserved motifs: Walker A, Q-loop, Walker B, D-loop, switch H-loop, and a signature motif (LSGGQ). The D-loop primarily functions in holding dimers together, the switch H-loop interacts with the transmembrane domain, the Walker A and B motifs form the P-loop, which binds to ATP, and the Q-loop and H-loop contain residues important for interacting with the γ-phosphate of the ATP [3]. Finally, the signature motif (LSGGQ) is exclusively found in ABC proteins, which enables ABC proteins to be distinguished from other ATPases [7].

In plants, a full inventory of ABC transporters was first catalogued for *Arabidopsis thaliana* [8], partially characterized [9, 10]. The current and most widely used classification system for ABC transporter subfamilies in plants is based on protein solubility, presence of TMDs, function, and amino acid sequence [5]. In our current study we used the system described in [5], which is consistent with the Human Genome Organization (HUGO) designation but including another subfamily, subfamily I. Plant ABC transporters fall into eight subfamilies using this nomenclature: A, B, C, D, E, F, G, and I (Table 1). ABCH subfamily members, a ninth subfamily, have not been identified in plants. Soluble proteins lacking TMDs such as the ABCF and ABCE members of the ABC transporter protein superfamily, despite being called such, lack any transport phenomena [5]. Despite the obvious importance of ABC transporters in plants, relative to other organisms there is a dearth of functional studies on individual members in plant as well as –omics studies; typically studies are limited to a few dicot species [8–14].

**Table 1:** A comparison of different nomenclature systems for ABC proteins

HUGO subfamily	Sánchez-Fernández subfamily^a^	ABCISSE family^b,c^	ABCISSE subfamily^b,c^	TC subfamily^d^	Domain organization	Taxa
ABCA	ABC1 homologue (AOH)	Drug and antibiotic resistance (DRA)	ABCA	Cholesterol/phospholipid/retinal flippase (CPR)	(TMD-NBD)2	eukaryotes (not yeast)
	ABC2 homologue (ATH)				TMD-NBD	eukaryotes
ABCB	Multidrug resistance (MDR)	Drug, peptides and lipid export (DPL)	p-glycoprotein (p-GP)	MDR	(TMD-NBD)2	prokaryotes and eukaryotes
	Transporter associated with antigen processing (TAP)		TAP and multidrug resistance-like protein (MDL)	TAP and mitochondrial peptide exporter (MPE)	TMD-NBD	eukaryotes
	ABC transporter of the mitochondria (ATM)		Heavy metal tolerance (HMT)	HMT	TMD-NBD	eukaryotes
	-		Lipid A-like exporter, putative (LLP)	-	TMD-NBD	prokaryotes and plants
ABCC	Multidrug resistance associated protein (MRP)	Organic anion conjugates and drug export (OAD)	MRP	Conjugate transporter (CT)	(TMD-NBD)2	eukaryotes
ABCD	Peroxisomal membrane protein (PMP)	Fatty acid export (FAE)	-	Peroxisomal fatty acyl-CoA transporter (P-FAT)	TMD-NBD; (TMD-NBD)2	bacteria and eukaryotes
ABCE	RNase L inhibitor (RLI)	RNase L inhibitor (RLI)	-	-	NBD-NBD	archaea and eukaryotes
ABCF	General control non-repressible (GCN)	Antibiotic resistance and translation regulation (ART)	Gene expression regulation (REG)	-	NBD-NBD	bacteria and eukaryotes
ABCG	White-brown complex homologue (WBC)	Eye pigment precursors and drugs (EPD)	WHITE	Eye pigment precursor transporter (EPP)	NBD-TMD	bacteria and eukaryotes
	Pleiotropic drug resistance (PDR)		PDR	PDR	(NBD-TMD)2	plants and fungi
ABCH	-	Drug resistance, bacteriocin and lantibiotic immunity (DRI)	YHIH		NBD-TMD	prokaryotes, slime moulds, echinoderms, insects and fish

References: ^a^[8], ^b^[6], ^c^[4], ^d^[52], Figure reprinted from [5] with permission

There are several known functions of ABC transporters of agricultural importance. One of the most important is detoxification. Detoxification mechanisms are of particular interest in herbicide resistance studies, particularly those involving putative glyphosate sequestration into vacuoles [15–17]. The *Arabidopsis* pleiotropic drug resistance 9 (PDR9) is also known to confer auxinic herbicide resistance [18]. Export proteins are also involved in environmental response and plant development such as auxin exporters ABCB19 and ABCB1 [19]. Importers primarily function in the acquisition of substrates important for cellular activity and ultimately the plant’s survival. Such is the case of AtPMP2, which imports critical substrates needed in the glyoxylate cycle during germination [20].

The main objective of our study was to survey the diversity of ABC transporters across plants using a new public resource for plant transcriptomes, the One Thousand Plants Transcriptome Project (1KP). 1KP includes raw transcript data as well as assembled reference transcriptomes generated with standardized processing procedures. These data included transcriptomes from two species with extant reference genomes: *Ricinus communis* (castor bean) and *Linum usitatissmum* (flax), which acted as internal controls for matching the 1KP transcriptomes with the published reference genomes. These resources offer a source of transcriptome sequencing data spanning the breadth of diversity of plants [13, 21–24].

## Methods

### Data acquisition and quality control

All of the data in this project, including raw sequence reads, transcriptome assemblies, and assembly statistics, were gathered from the 1KP collaborative project [21]. The 1KP consortium performed all sample collection, sequencing, quality control, and assembly [13, 21]. A total of 1462 assemblies, assembled with SOAP de novo, were downloaded from the 1KP repository [11, 25]. Because some crucial taxonomic information was missing from accompanying data files, 161 samples were excluded from subsequent analysis. Another six samples were removed because of contamination that was discovered during the quality control step. The remaining 1295 1KP samples were translated into six frames using the Transeq program from EMBOSS (The European Molecular Biology Open Software Suite) [26]. A six-frame translation coupled with searches amongst confirmed ABC transporter protein sequences enabled us to assign each transcript into the correct reading frame for analysis. Since well-annotated genomes exist for only a handful of species analyzed and most species lacked sufficiently large and diverse tissue sequencing results, alternative splice variants were not considered.

### HMMER domain e-value selection

The full sets of *Arabidopsis* and rice protein sequences were obtained from Phytozome version 10, including the *Arabidopsis* ABC transporter protein list [5] and rice list [27] Additional file 1. The hmmscan program from the HMMER package version 3.1 was used to search for the pfam record PF00005 across the full *Arabidopsis* and rice protein data sets [5, 28, 29]. A pfam record, such as the PF00005 domain, is a hidden Markov model based on a multiple sequence alignment of protein domains; in the case of PF00005 this multiple sequence alignment is of the NBD of many different ABC transporter proteins. A cutoff e-value of 3.10E-18 was selected in the two data sets that maximizes true positives (reported actual ABC transporters) and minimizes false negatives (reported as non-ABC transporters).

### Comparison of genome and transcriptome data sources

The full set of flax and castor bean proteins were obtained from Phytozome v10 [30, 31]. Three sets of transcriptomic data for flax (shoot sample 1, shoot sample 2, shoot sample 3) and one set of castor bean (mixed tissue sample) were obtained from the 1KP data set [11, 25]. The hmmscan program was used to search for PF00005 pfam domains among all six sets of data using an e-value of 3.10E-18, yielding six sets of putative ABC transporter genes.

### ABC transporter classification

Each sample’s translated transcriptome was searched using hmmscan [28]. Search results containing the PF00005 pfam domain with an e-value of 3.10E-18 were subjected to further classification using best BLAST searches [32]. The de novo assembled transcriptomes from the 1KP data set were fragmented, transporters containing multiple nucleotide binding domains may only have had one of their binding domains accounted for in the transcriptomes sequencing results. Determination of ABC transport protein family was made strictly using BLAST searches against known ABC transport proteins in Arabidopsis and rice. A specialized database of known plant ABC transporters from *Arabidopsis* and rice was constructed for subfamily classification purposes using legacy BLAST formatdb version 2.2.26 (Additional file 2: Table S1). All known plant ABC transporter subfamilies were represented in our BLAST database.

Each of the 1295 1KP subsamples along with flax and castor bean genome-derived proteomes were used to query the ABC transporter database using legacy BLAST version 2.2.26, in each case the best hit to the ABC transporter database was used to classify the query among one of the eight ABC transporter subfamilies.

### Nonparametric comparison of ABC diversity

JMP version 11 [33] was used to perform a one-way analysis of counts by species and performed pairwise nonparametric comparisons using the Wilcoxon method. All subfamily and total ABC transporter gene member classifications were subjected to pairwise analysis between each of the groups: angiosperms, conifers, ferns, lycophytes, mosses, hornworts, liverworts, chlorophytes, charophytes, glaucophytes, rhodophytes, and Chromista.

## Results and discussion

### ABC protein discovery

The pfam record PF00005 represents the highly conserved ATP-binding domain of ABC transporters. We used this pfam record to identify ABC transporter gene members across an entire transcriptome database as well as full gene sets of *Arabidopsis* and rice (Table 2). ABC transporter protein sequences had much lower expectation values for the PF00005 pfam domain from the hmmscan than did known non-ABC transporter protein sequences (85 % of the top 200 lowest e-value hits were known ABC transporter proteins) (Additional file 3: Table S2). These results were obtained using 233 known ABC transporters and 85,152 known non-ABC transporter protein sequences. While the pfam search was effective in ABC transporter gene member discovery, there was not a clear delineation in e-value scores between known gene family members and other genes. The highest e-value among known ABC transporters was 2.10E-10. However, some non-ABC transporters showed e-values lower than the 2.10E-10 cutoff. Searching among the top five highest e-values we identified the largest gap between e-values of 3.10E-18 and 2.10E-15. This gap represents three orders of magnitude between non-ABC transporters and ABC transporters. Using the 3.10E-18 threshold of significance, we identified 282 sequences as ABC transporters. Of the 282 sequences identified as ABC transporters, 230 had been previously classified as ABC transporters, but 52 were not (putative false positives). Therefore, the cutoff excluded three known ABC transporters (putative false negatives). With the entire set of 85,152 starting sequences, the usage of the pfam PF00005 domain yielded a sensitivity (true positives divided by true positive plus false negatives) of 0.9871 and a specificity of 0.9994 (true negatives divided by true negatives plus false positives). Consequently, an e-value of 3.10E-18 was selected as the optimum, which allowed a total of three false negatives for ABC transporter detection for all further HMMER analysis.

**Table 2:** A summary of HMMER search results

E-value cutoff	True positives	False positives	True negatives	False negatives	Specificity	Sensitivity
3.10E-21	222	48	84,882	11	0.9994	0.9528
3.10E-18	230	52	84,870	3	0.9994	0.9871
2.10E-15	231	52	84,869	2	0.9994	0.9914
2.10E-13	232	55	84,865	1	0.9994	0.9957
2.10E-10	233	64	84,855	0	0.9992	1.0000

Summary of sensitivity and specificity of 85,152 HMMER queries against pfam as a method for selecting an appropriate cutoff e-value for the identification of PF00005 (ABC transporter) domains

### Comparison of genome and transcriptome data sources

The flax and castor bean transcriptomes allowed us to examine how the number of expressed ABC transporters identified in 1KP corresponds to the number of known ABC transporters in their entire genomes, which allowed us to make inferences throughout the plant kingdom, with caveats. ABC transporter genes are known to have variable expression across tissues [11], and thus their identification from RNA-Seq data is unlikely to yield a complete complement of gene family members for an organism.

The 1KP-generated assemblies for flax had 99,855, 95,813, and 101,110 total putative transcripts for each library. The number of transcripts identified by hmmscan as ABC protein transporter gene members was similar between the three flax samples: 82, 91, and 92. These figures represented 42 – 47 % of the number of ABC transporters identified from the reference genome protein sequences. A total of 59 transcripts were putatively identified as ABC transporters from the single available castor bean transcriptome from 1KP, 42 % of the 142 putative genes identified in the genome-sequence derived gene set.

**Fig. 1:**
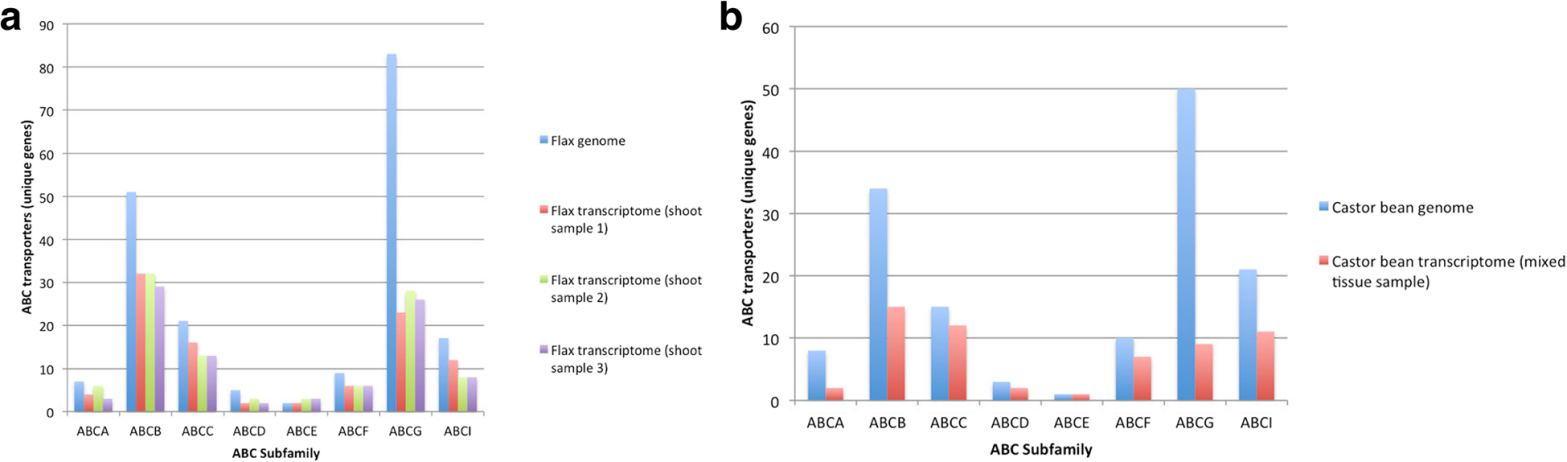
Flax and castor bean ABC transporter unique gene distribution inferred from RNA-Seq transcriptome data. **a** Plot comparing total ABC transporter unique genes observed in the flax genome and the three 1KP transcriptomes for flax (shoot sample 1, shoot sample 2, shoot sample 3). **b** Plot comparing total ABC transporter unique genes observed in the castor bean genome and the 1KP transcriptomes for castor bean (mixed tissue sample). Searches based on hmmscan searches for the PF00005 pfam domain and subsequent BLAST queries against a custom BLAST database of ABC transporters from *Arabidopsis* and rice

Subfamilies for putative ABC transporters were assigned on the basis of the most similar amino acid sequence from *Arabidopsis* or rice. The distribution of the ABC transporters among the eight subfamilies shared a similar pattern between the 1KP transcriptome samples and the reference genome-derived gene sets (Figs. 1a and b).

In general, the 1KP data per accession contained nearly half the number of unique ABC transporter gene members, by comparison, to their reference genome counterparts as identified by hmmscan searches. In addition, hmmscan found nearly the same number of unique ABC transporters for each of the three 1KP data sets for flax. The relative consistency between corresponding 1KP data samples is encouraging for extension of inferences across data sets in order to estimate the total number of gene family members for all plant taxa.

### ABC transporter subfamily classification

The majority of our knowledge about the ABC transporter protein superfamily in plants is from studies limited to a few angiosperm species. In an effort to expand our knowledge of the varying representation of ABC transporter protein subfamilies across the plant kingdom, we classified nearly 97,149 transcripts across 1295 samples into one of the seven ABC transporter HUGO subfamilies (A, B, C, D, E, F, G) and the plant-only subfamily, I [5]. The 1295 1KP samples span a diversity of plant taxa: 816 from angiosperms, 76 from conifers, 71 ferns, 22 lycophytes, 25 liverworts, 41 mosses, 6 hornworts, 123 chlorophytes, 46 charophytes, 5 glaucophytes, and 28 rhodophytes. In general, the results comparing hornworts/glaucophytes and other plants were not significant, likely owing to the small sample sizes in these groups. The breadth of samples across various plant groups provides robust statistical support for comparisons amongst groups in most cases. The gymnosperms were mainly represented by conifers, with few other gymnosperms being represented: three Gnetales, four Cycadales, and one Ginkgoales.

**Fig. 2:**
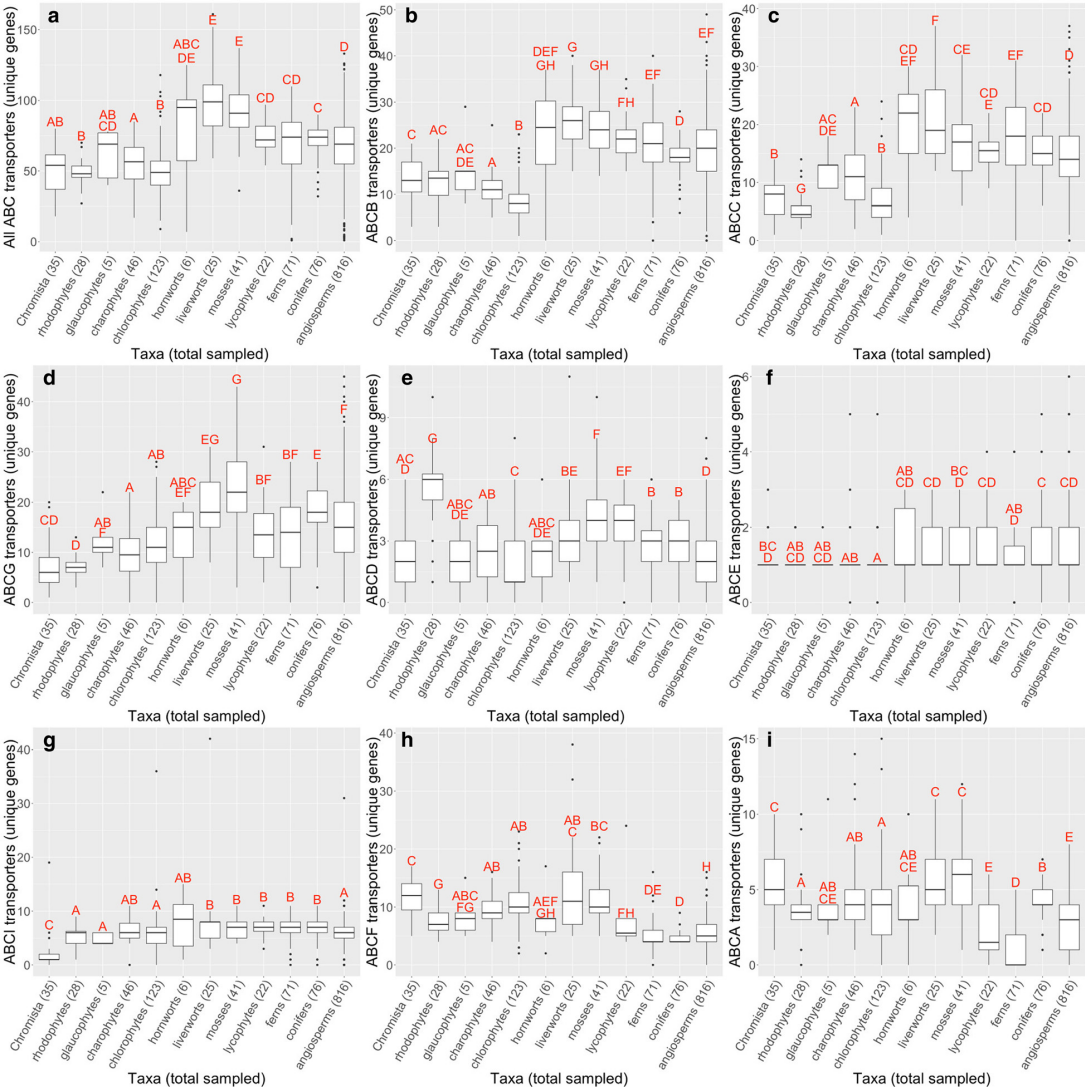
Number of unique ABC transporter gene members distributed over plant taxa inferred from RNA-Seq transcriptome data. **a** Box and whisker plot of subfamily ABC transporter unique genes among each set of samples in indicated plant groups: Chromista, rhodophytes, glaucophytes, charophytes, chlorophytes, hornworts, liverworts, mosses, lycophytes, ferns, conifers, and anigosperms. **b** ABCB transporter gene members. **c** ABCC transporter gene members. **d** ABCG transporter gene members. **e** ABCD transporter gene members. **f** ABCE transporter gene members. **g** ABCI transporter gene members. **h** ABCF transporter gene members. **i** ABCA transporter gene members. Classifications are based on hmmscan searches for the PF00005 pfam domain. Significant differences between unique gene counts of the twelve groups were determined by a comparison of means using the Wilcoxon method. Filled circles indicate outliers. The total samples per plant group are indicated in parentheses beside each label along the x-axis. Statistical differences are indicated by letter groupings (*p* < 0.005)

The hmmscan analysis with a cutoff of 3.10E-18 found at least 50 unique ABC genes (transcripts) containing PF00005 pfam domains per plant sample (Fig. 2a). In many cases, we recorded outliers, the largest of which, *Blasia sp*. (526 ABC transporters), more than doubled the next largest sample. For ease of interpretation, the *Blasia sp*. sample was omitted from figures in most cases. Bryophytes, lycophytes, conifers, ferns, and angiosperms had 16 to 43 more ABC transporter transcripts than rhodophytes, chlorophytes, charophytes, glaucophytes, and Chromista. Many observed differences in average number of transporters between plant groups were found to be significant (*p* < 0.005). Although green algae and land plants have been shown to harbor similar numbers of ABC transporter subfamily gene members and total ABC transporter genes [34], the simpler transport systems of green algae, rhodophytes, glaucophytes, and Chromista may contribute to the reduced number of unique ABC transporter genes we observed on our study. Angiosperms, conifers, ferns, chlorophytes, charophytes, glaucophytes, rhodophytes, and Chromista had 22 to 43 fewer unique ABC transporter gene members than bryophytes. The differences in mean number of transporters between these groups is also significant, with exception to hornworts (*p* < 0.005).

Results from BLAST subfamily classifications were used to identify full-length transcripts based on query coverage. Because the ABC transport protein subfamilies split prior to the most recent common ancestor of all plants, it is likely that ABC transport protein subfamily members will closely match an ortholog of the same subfamily. Transcripts with greater than 90 % of the query aligned to the target sequence were classified as full length. All 1295 transcriptome samples contained at least one putative unique ABC transporter gene member with 97,149 ABC transporters identified across all samples. A total of 22,343 ABC transporter sequences were identified as full length. There were, on average, 66 ABC transporter gene members discovered per RNA-Seq library with a standard deviation of 26 gene members (Fig. 2a). Most samples (1052 of 1295) contained at least one of each of the ABC transporter protein subfamily members.

The number of unique ABC transporter subfamily gene members varied between distantly related plants. Among the functionally diverse subfamilies ABCB and ABCC, we saw significantly fewer unique ABC transporter gene members on average in algae (5 to 15 gene members) than in other groups: angiosperms, conifers, ferns, lycophytes, and bryophytes (*p* < 0.005) (Figs. 2b and c). Among ABCG subfamily transporters the differences in the average number of ABC transporter gene members seen between algae was significantly lower (5 to 11 gene members) than the following groups: angiosperms, conifers, and bryophytes (*p* < 0.005) (Fig. 2d).

Several studies have indicated that plant genomes have more ABCB, ABCC and/or ABCG subfamily gene members in comparison to other eukaryotes such as human and yeast [5, 35]. Our results revealed additional variability among plants regarding the size of these subfamilies, specifically in that algae contain relatively fewer unique ABCB, ABCC and ABCG transporter members. The larger number of unique ABCB transporter gene members seen in vascular plant species may have adaptive significance for complex and heterogeneous environments of “higher plants.” Land plants’ best defense against xenobiotics, such as heavy metals, might come from ABCB transporters’ ability to sequester and/or transport foreign chemicals. For example, AtABCB25, an *Arabidopsis* ABCB export protein, allows some tolerance to cadmium and lead when overexpressed [36, 37]. The overexpression data are useful to infer functional significance, but also guide potential utility in biotechnology. Aluminum tolerance is conferred by the expression of ABCB27 in root tips [38]. ABCC transporter proteins have been identified among plants for their role in detoxification and the regulation of stomatal guard cells [39]. The gap in types of ABCC transporter transcript number seen between algae and other plant taxa might be explained by a lack of need by algae for stomata regulation and/or detoxification. While liverworts also lack stomata we do not see significantly fewer ABCC transporter proteins among liverworts. Algae do not require the specialized transport systems of land plants because most cells have direct access to nutrients in their water environment. Such direct access to an aquatic environment may contribute to fewer unique gene members of subfamilies ABCB and ABCC. Only 26 ABC transport proteins were found in *Ceratophyllum demersum,* relative to average 66 ABC transport proteins seen across all 1295 tissues sampled. This finding would appear to support the reduced expression of ABC transport proteins in an aquatic environment (Additional file 4: Table S3). Algae also do not produce a waxy cuticle - so reduced ABCG transcripts may be related to a lack of the ABCG transporter proteins that are necessary for the transport of cuticular lipids [40, 41]. Whether the variation in size of these families is a result of gene expansion is unclear and requires further investigation for evaluation.

Among subfamilies ABCD, ABCE, and ABCI, few significant differences were observed in the average number of gene members across plants with a single exception in the ABCD subfamily (Figs. 2e, f, and g). Significant differences in the average number of ABC transporter gene members detected among ABCD, ABCE, and ABCI were small, ranging from zero to two (*p* < 0.005). The presence of ABCD, ABCE, and ABCI transcripts with very similar gene member averages might suggest that the proteins that result from the translation of these transcripts are essential to life among all plants. Plant ABCD transporters are well known for their import of substrates necessary in the glyoxylate cycle [20]. It seems likely that plants retaining ABCD transporters would have an evolutionary advantage over plants that do not, given the importance of the glyoxylate cycle to survival across all plant taxa. Rhodophytes have nearly double, on average, the number of ABCD subfamily members compared with other plants. We can speculate that since these ABCD transporters are peroxisome-localized and important for fatty acid metabolism, especially long chain fatty acids, and that Rhodophyta produce a high number of long-chain fatty acids [42], that there may be functional significance. Rhodophytes might be good targets to mine ABCD transporters for use to produce novel oils in crops. ABCE proteins are found in archaea, bacteria, and eukaryotes, suggesting that this class of ABC transporter proteins is essential to life and are thus conserved [5]. ABCE1 is a translation termination factor [43]. Once a ribosome reaches a stop codon, ABCE1 helps to detach the ribosome from the mRNA [44], and also plays an important role in ribosome recycling [45]. The maintenance of similar numbers of unique ABCD and ABCE subfamily gene members may be because of the conservation of essential functions provided by those subfamilies. Our understanding of the ABCI subfamily, is expected to be significantly improved as more information regarding the structure and function of the subfamily members becomes available. However, current data suggest the origins of ABCI subfamily transporter genes may be from prokaryotic genomes and so they may result from a movement of genes from mitochondria and plastids to the nuclear genome. As such, the ABCI transporter proteins have been classified as a heterogeneous group composed of multicomponent transporters (reviewed in [5]) so the similarity seen in average number of unique gene members across plant taxa was unexpected.

In the case of ABCF subfamily proteins, we observed significantly higher transcript count averages in Chromista, glaucophytes, rhodophytes, chlorophytes, charophytes, and bryophytes than in all other groups, four to seven more transcripts on average than angiosperms, conifers, ferns, and lycophytes (*p* < 0.005) (Fig. 2h). When directly comparing algae and bryophytes, we found that bryophytes had more unique ABCF subfamily gene members on average (*p* < 0.005). Understanding the increased variation, specifically regarding the ABCF subfamily size among algae and bryophytes is difficult with a lack of functional characterization of ABCF proteins among plants. Studies in yeast and humans have indicated ABCF protein function in the activation of eIF-2α kinase [5]. Activation of eIF-2α kinase is important in the regulation of stress response factors [46]. Indeed one member of the ABCF, ABC50 appears to play an especially-important role in mammalian translation processes in interactions with eiF2 and the ribosome [47, 48]. There is a published patent application in which ABC50 is claimed to be useful to increase protein in plants, among other organisms [49]. We noted an increase in the total number of ABCF protein gene members among algae and bryophytes; we cannot speculate about the potential adaptive significance.

The average number of unique ABCA subfamily transporter gene members was significantly lower in ferns, three to four less gene members on average in most cases (*p* < 0.005) (Fig. 2i). There was a significant difference between the average number of unique ABCA transporter gene members in ferns, conifers, and angiosperms (*p* < 0.005). While little is known about the function of ABCA transporter proteins among plants, they are suspected to be involved in lipid metabolism based on their known function in humans (reviewed in [50]). Upon comparison with *Arabidopsis*, rice was found to be missing an orthologous full length ABCA transporter, which is consistent with previously published results [51]. Our results indicate an occurrence of decreased ABCA transporter genes among ferns relative to other plant groups, possibly from gene loss. While the single gene loss observed in rice and the results observed in fern are likely unrelated, they may point to similar phenomena regarding the importance of ABCA transport proteins in sterol metabolism [52].

## Conclusions

ABC transport proteins have diverse functions and are ubiquitous in plants. We catalogued ABC transporters across the plant kingdom from hundreds of transcriptomes. While our current study was constrained by imperfect transcriptome assemblies taxonomic patterns should be of interest to the community to build hypotheses. Specifically, we found significant differences in the number of unique ABC transporter gene members between algae (Chromista, rhodophytes, glaucophytes, chlorophytes, and charophytes), bryophytes (mosses, liverworts, hornworts), lycophytes, ferns, conifers (other gymnosperms were not well represented among the 1KP data), and angiosperms. Algae and bryophytes have fewer unique ABC transporter gene members than lycophytes, ferns, conifers, and angiosperms. The number of unique ABCD, ABCE, and ABCI subfamily protein gene members show few significant differences amongst plants. With regards to plant biotechnology applications, it is noted that these three subfamilies might not provide much novelty. In contrast, the diversity of ABCB and ABCF proteins, known for detoxification and stress resistance, respectively, are attractive genes to screen for improving crops via biotechnology. Especially intriguing are the ABCF members in algae and bryophytes for potential use in crop biotechnology. Given the diversity and function of ABCF members, algae and bryophytes have been largely ignored as sources of potentially useful genes in agriculture.

## Additional files

Additional file 1: file S1. Fasta file of ABC transporter proteins from rice and *Arabidopsis*. (FASTA 139 kb) Additional file 2: Table S1. Detailed table of HMMER testing results. (XLSX 82 kb) Additional file 3: Table S2. Detailed table of classification results and associated metadata. (XLSX 163 kb) Additional file 4: Table S3. Filtered BLAST results of transcripts with 90 % or greater query coverage of ABC transporter protein subject. (XLSX 2317 kb)
